# Increased plasma homocysteine levels in patients with multiple sclerosis and depression

**DOI:** 10.1186/1744-859X-7-17

**Published:** 2008-09-09

**Authors:** Nikolaos Triantafyllou, Maria-Eleftheria Evangelopoulos, Vasilios K Kimiskidis, Evangelia Kararizou, Fotini Boufidou, Konstantinos N Fountoulakis, Melina Siamouli, Chrysoula Nikolaou, Constantinos Sfagos, Nikolaos Vlaikidis, Dimitrios Vassilopoulos

**Affiliations:** 1 Department of Neurology, Eginitio Hospital, University of Athens, Greece; 2 Department of Neurology III, Aristotle University of Thessaloniki, Greece; 3 Department of Psychiatry III, Aristotle University of Thessaloniki, Greece

## Abstract

**Background:**

The aim of the study was to assess the plasma levels of homocysteine in patients with multiple sclerosis (MS) and to investigate whether an association with depression exists.

**Methods:**

Plasma homocysteine (Hcy), vitamin B12 and plasma folate were measured in 65 moderately disabled patients with relapsing/remitting MS (RR-MS) and 60 healthy controls. All subjects were assessed with the Beck Depression Inventory (BDI).

**Results:**

Hcy levels were significantly increased in MS patients compared to controls (13.5 ± 4.7 μmol/l vs 8.5 ± 3.1, p < 0.001). A significant correlation was found between Hcy levels and BDI scores (Pearson r = 0.3025, p < 0.05). Plasma Hcy was not related to Extended Disability Status Scale (EDSS) score, age, disease duration or vitamin B12 and folate.

**Conclusion:**

Moderately disabled MS patients with elevated Hcy levels are particularly prone to develop depressive symptomatology. Further study is warranted in order to elucidate the prognostic and therapeutic implications of this novel finding.

## Background

Homocysteine is a non-essential sulfur-containing amino acid derived from methionine by demethylation. Vitamins B12 and B6 as well as folate play an important role in the metabolic pathway of homocysteine [1]. A number of recent studies support the notion that homocysteine might cause neuronal damage by triggering oxidative injury and DNA damage [2-5]. As a result, homocysteine has been implicated in the pathogenesis of numerous neurodegenerative and psychiatric disorders [1].

Amongst neurological conditions, multiple sclerosis (MS) has been extensively investigated with regard to homocysteine metabolism, with conflicting results. Some studies demonstrated elevated plasma homocysteine (Hcy) levels in MS patients with [6,7] or without B12 deficiency [8,9]. By contrast, a single report found no difference in Hcy levels between MS patients and controls [10].

With regard to psychiatric disorders, depression has been linked in particular to increased Hcy plasma levels in the context of altered methylation reactions [11-13]. No study, however, has attempted to address these two issues jointly; that is, to investigate plasma homocysteine levels in patients with MS and depression.

Accordingly, the present study was designed in order to assess Hcy plasma levels in MS patients and investigate whether an association with depression and clinical disability or disease duration exists.

## Methods

A total of 65 relapsing remitting MS (RR-MS) patients with a mean age 39.2 ± 8.3 years, entered the study after providing informed consent for the procedures. All patients met the criteria for clinically definite MS according to Poser *et al*. [14], were in remission and had not received treatment with corticosteroids for at least 2 months prior to their inclusion in the study. The mean duration of the disease was 9.8 ± 5.9 years and disability according to Extended Disability Status Scale (EDSS) was 3.2 ± 1.7 points. None of the patients had received B12 or folate supplementation. All patients included in the study had normal blood pressure, lipid profile and renal function and had no risk factors for atherosclerosis.

A total of 60 healthy volunteers with a mean age of 38.2 ± 7.7 years served as a control group. The demographic characteristics of MS patients and healthy controls are shown in Table 1.

**Table 1:** Demographic characteristics of multiple sclerosis (MS) patients and controls

	**RR-MS**	**Controls**
No. of subjects	65	60
Age	39.2 ± 8	38.2 ± 7.7
Sex (F/M)	40/25	40/20
Disease duration	9.8 ± 5.9	-
EDSS	3.2 ± 1.8	-

EDSS, Extended Disability Status Scale; Hcy, plasma homocysteine; RR-MS, relapsing/remitting MS.

After overnight fasting, blood samples were drawn from a peripheral vein and were kept on ice prior to separation (maximum 30 min after drawing). Total homocysteine (free plus protein-bound) was quantified using a sandwich enzyme immunoassay method based on a monoclonal/polyclonal antibody pair (Axis-Shild Diagnostics, Dundee, UK). Values higher than 15 μmol/l were considered increased according to relevant literature and manufacturer's instructions.

Serum folate and B12 levels were quantified with a paramagnetic particle chemiluminescence's immunoassay (Access 2 immunoassay system, Beckman Coulter, Nyon, Switzerland).

Assessment of depressive symptomatology in all subjects was performed by the Beck Depression Inventory (BDI) [15], a self-report screening instrument for depression. The questionnaire consists of 21 items that measure the severity of depression in adults scored from 0 to 63. Subjects with BDI scores greater than 13 were characterised as depressed [16,17]. According to the Goldman consensus statement on depression in MS, this cut-off level is satisfactory, in terms of sensitivity and specificity, in ambulatory patients with MS and was therefore appropriate in the context of the present study [16].

### Statistics

Statistical testing was performed by Fisher's exact test for categorical variables whereas an unpaired t test was used for comparing continuous variables. Correlations between Hcy levels and other investigated parameters were derived using Pearson correlation analysis. The level of statistical significance was set at p = 0.05. All statistical analyses were performed with SPSS version 10.0 for Windows (SPSS Inc., Chicago, IL, USA).

## Results

**Figure 1:**
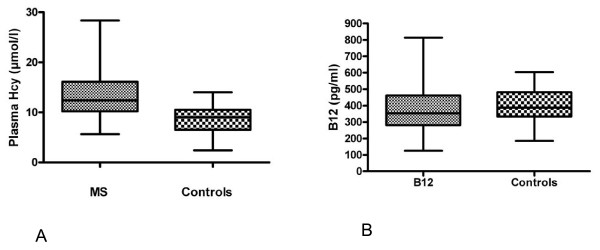
**Box and whisker plot of plasma homocysteine (Hcy) and vitamin B12 in multiple sclerosis (MS) patients and controls**. Hcy, but not B12 levels, were significantly different amongst the two groups (p < 0.001).

Plasma Hcy levels were significantly increased in MS patients compared to controls (13.5 ± 4.7 μmol/l vs 8.5 ± 3.1, p < 0.001; Figure 1A). This increase occurred in the absence of significant differences in the serum levels of folate and B12 between MS patients and controls (Figure 1B). Using a cut-off level of 15 μmol/l, MS patients were further subdivided into those with normal Hcy (values = 15 μmol/l) and patients with increased Hcy (> 15 μmol/l). MS patients with increased Hcy levels had higher BDI scores compared to patients with low Hcy, (18.4 ± 14.4 vs 9.3 ± 8.5, respectively, p < 0.05) (Table 2). In addition, the prevalence of depression was higher in the MS group with increased Hcy (8 out of 18,44%) compared to patients with normal Hcy (8 out of 47,17%, p = 0.05).

**Table 2:** Demographic and clinical characteristics of RR-MS patients classified according to Hcy levels

	**Increased Hcy RR-MS**	**Normal Hcy RR-MS**
Age	41.8 ± 10.0 *	37.8 ± 7.0
Disease duration	10.7 ± 6.4†	9.621 ± 5.3
EDSS	4.2 ± 2.2†	3.1 ± 1.4
BDI	18.40 ± 14.4§	9.39 ± 8.3

*p = 0.03; †p = NS; §p = 0.01.

BDI, Beck Depression Inventory; EDSS, Extended Disability Status Scale; Hcy, plasma homocysteine; RR-MS, relapsing/remitting MS.

**Figure 2:**
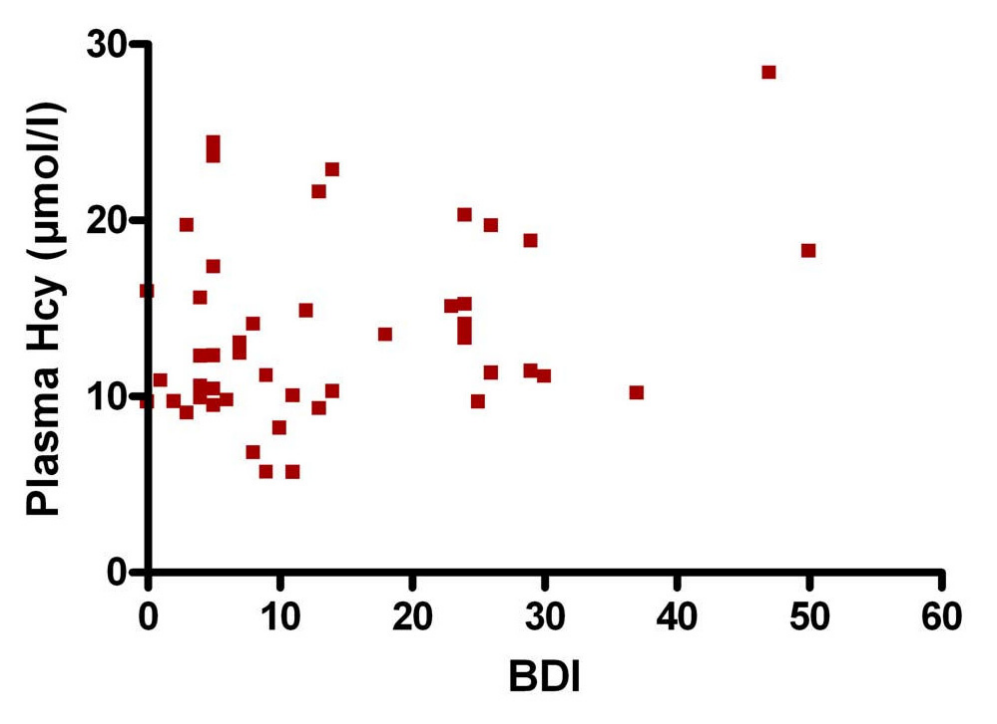
Correlation graph between plasma homocysteine (Hcy) and Beck Depression Inventory (BDI) scores.

Pearson correlation analysis showed that BDI was significantly associated with Hcy (r = 0.3025, 95% CI = 0.01670 to 0.5426 p < 0.05; Figure 2). By contrast, Hcy concentrations were not significantly related to other investigated variables (age, disease duration, EDSS, vitamin B12 and folate) (Table 3).

**Table 3:** Correlations of homocysteine with disease duration, age, EDSS and BDI scores, vitamin B12 and serum folate

	**Pearson r**	**p Value**
BDI	0.3	0.04 *
Disease duration	0.01	0.9
EDSS	0.9	0.5
Age	0.02	0.8
Vitamin B12	0.042	0.7
Folate	-0.65	0.68

BDI, Beck Depression Inventory; EDSS, Extended Disability Status Scale.

## Discussion

The present study addressed the issue of Hcy metabolism in MS and investigated whether Hcy levels are related to depression in this disease. It is concluded that Hcy is increased in patients with RR-MS without concomitant signs of vitamin B12 and folate deficiency. In addition, we report, for the first time, a positive correlation between increased Hcy levels and depressive symptomatology in MS patients.

Previous studies regarding Hcy metabolism in MS have provided contradictory results. With a single exception [10], these studies concluded that Hcy levels are increased in MS patients [8,9]. In most cases, however, Hcy increases were observed in MS patients with vitamin B12 deficiency [7,18,19]. Our results, in agreement with Vrethem *et al. *[8] and Ramsaransing *et al. *[9], indicate that Hcy increases may occur in MS in the absence of vitamin B12 and folate deficiency.

Of particular interest is the fact that MS patients with increased Hcy had higher BDI scores compared to patients with normal Hcy. From a methodological point of view, it should be pointed out that BDI, in common with other depression screening instruments, has certain limitations in the evaluation of MS patients due to a considerable overlap between the signs and symptoms of depression and the signs and symptoms of MS. As a result, BDI may overestimate depression among patients with somatic complaints, for instance fatigue [20] which typically occurs in MS. By contrast, BDI represents a reliable tool with high internal consistency in the evaluation of depression [21].

Depression occurs frequently during the course of MS and is multifactorial in origin. It is associated with the degree of clinical disability, as reflected by the EDSS score [22-24] and the particular subtype of the disease [25] although these findings were not replicated in all studies [22,26].

What is particularly relevant to our study is the fact that severe depression is associated with increased Hcy levels [27]. A recent population-based study confirmed this link by showing that subjects with increased Hcy levels are more likely to suffer from depression [11] while other studies indicated that folate deficiency and the MTHFR C677T polymorphism may be causally related to depression [12,13].

At the biochemical level, the rise in homocysteine levels observed in patients with depression is ascribed to failure of methylation of Hcy to methionine due to a shortage of supply of methyl groups from methyl folate or lack of the vitamin B12 cofactor for this methylation reaction [27]. Methionine, in turn, is the precursor of *S*-adenosylmethionine, the methyl donor in a host of methylation reactions in the central nervous system (CNS) involving monoamines and various neurotransmitters, amongst other cellular constituents. Thus, increased Hcy levels in depression are thought to reflect functional folate and/or B12 deficiency, which may ultimately cause an imbalance at the monoamine or neurotransmitter level.

The mechanism of increased Hcy levels in MS patients, particularly those with depression, seems to be different since folate and/or B12 deficiency was not documented in the context of the present study. Irrespective of the precise pathogenetic mechanisms, however, increased Hcy may induce neuronal damage through its neurotoxic action [2-5]. It is also possible that raised Hcy may accentuate CNS inflammatory processes by decreasing the production of apolipoprotein A1 (apoA-I) [28]. ApoA-I is proposed to display anti-inflammatory properties by interfering with monocyte-lymphocyte interaction and inhibiting tumour necrosis factor (TNF)α and interleukin (IL)1β production [29,30].

The clinical implications of our finding are not entirely clear. Since Hcy is associated with an atherogenic propensity, MS patients with elevated Hcy and depression might represent a subgroup of MS patients with a predisposition for atherothrombotic cerebrovascular disease. In addition, since our patients were not entered into a treatment study, it is currently unknown whether vitamin treatment combined with antidepressants is the optimal way to treat this subgroup of MS patients.

In conclusion, our results suggest that moderately disabled MS patients with elevated Hcy levels are particularly prone to develop depressive symptomatology, reflected by higher BDI scores. Further studies and follow up of this subgroup of MS patients are warranted in order to elucidate the prognostic and therapeutic implications of this finding.

## Competing interests

The authors declare that they have no competing interests.

## Authors' contributions

NT conceived of the study, and participated in its design and coordination, carried out the statistical analysis and drafted the manuscript. MEE and VKK participated in the design of the study and drafted the manuscript. EK and MS participated in data acquisition. FB and CN carried out the immunoassays. KNF participated in the design of the study and drafted the manuscript. CS, NV and DV participated in the interpretation of data. All authors read and approved the final manuscript
